# Does Suture Configuration Matter in Achilles Tendon Repairs?

**DOI:** 10.7759/cureus.94982

**Published:** 2025-10-20

**Authors:** Nathan Benner, Hana Keller, Grant Branam, Scott Telfer, Kenneth Chin

**Affiliations:** 1 Orthopaedics, University of Washington, Seattle, USA; 2 Orthopaedics, Washington State University, Pullman, USA; 3 Orthopaedic Surgery, University of Washington Medical Center, Seattle, USA

**Keywords:** achilles tendon, achilles tendon repair, sports medicine of the foot and ankle, sports problems, tendon biomechanics

## Abstract

Background: The human Achilles tendon is functionally important for dynamic activities and is a common site of tendon rupture, resulting in pain, weakness, and removal from sport. While treatment options vary and the opinions regarding optimal management are not uniform, percutaneous repair through commercially available guides is one option that has been used in the United States.

Hypothesis/purpose: The purpose of this study was to test the mechanical properties of four common suture configurations in the repair of Achilles tendon ruptures.

Study design: This biomechanical study utilized porcine toe flexor tendons as an analogue for the human Achilles tendon.

Methods: Simulated rupture was performed, and tendons were repaired using a percutaneous guide. The technique was uniform with predetermined allotment into one of four groups based on suture configuration (single or double locked) and type (round or flat), with a total of 10 specimens in each group. Specimens then underwent a static creep test, a dynamic load creep test, and finally a load-to-failure test. An analysis of variance was performed to test for differences between the variables, followed by pairwise comparisons using independent t-tests to assess inter-group differences if significant effects were found, adjusted for multiple comparisons.

Results: No significant differences were seen between conditions for the creep tests. The suture configuration used was determined to have a significant effect on the maximum load to failure of the constructs (p = 0.018) and maximum stress in the construct during the load-to-failure test (p = 0.019). Double tape had a significantly greater load to failure than the single round and single conditions and reached greater stresses before failure (respectively). No significant differences were found between the double tape and double round techniques, nor between the double round and either of the single techniques for any of the load-to-failure variables.

Conclusion: The results of this study, using a porcine model to simulate a minimally invasive technique for Achilles tendon repairs, suggest that the use of a double-locked tape suture configuration leads to a stronger overall construct.

## Introduction

The Achilles tendon is the largest and strongest tendon in the human body [1, 2, 3]. Despite this, it is one of the most commonly injured. An acute rupture of the Achilles tendon results in significant pain and weakness and, for the athlete, can result in removal from sport for an average of six months [4]. This injury has a reported annual incidence of 29.3/100,000 person-years and is at least twice as common in men [5]. The incidence of Achilles tendon injuries has increased, while the prevalence of surgical treatment has decreased from 16.9/10,000 in 1994 to 6.3/10,000 in 2013. This decline in the rate of surgical repair has been attributed to a landmark study from Willits et al., showing no difference when comparing operative to non-operative treatment of Achilles tendon repairs [6]. There are multiple prior randomized controlled trials and meta-analyses comparing nonoperative to operative treatment for Achilles tendon ruptures, with no clear consensus on treatment [7]. A recent article in the New England Journal of Medicine demonstrated a higher risk of re-rupture in patients treated without surgery than in those treated with open or minimally invasive surgery. There is also a reported decrease in overall heel lift-off strength at one year in patients treated nonoperatively [8]. Given these suggested benefits of surgery, and often by patient or surgeon preference, many patients undergo operative management of acute Achilles ruptures.

Broadly, surgical management of Achilles tendon ruptures is performed with open or minimally invasive techniques. Minimally invasive surgery consists of a small incision and sutures placed percutaneously through the Achilles tendon, often through commercially available guides. In recent years, this has become more popular with the trend towards minimally invasive surgery and concerns about wound complications with traditional open approaches [7].

In addition to choosing a surgical approach for tendon repair, one must consider which configuration of sutures will have the lowest rate of failure. It has been shown that the addition of locked sutures leads to a greater construct strength for both cyclic and ultimate load to failure [1]. There are no prior studies comparing round suture to suture tape or the use of double-locked suture to single-locked suture configurations. The purpose of this biomechanical study was to test the biomechanical strength of four suture permutations using the porcine flexor tendon as a model for the human Achilles tendon. We hypothesized that a double-locking configuration would be stronger than single locking and that suture tape would be stronger than round suture.

An electronic poster of this abstract was presented at the American Orthopaedic Foot & Ankle Society (AOFAS) 2023 annual meeting in Louisville, KY (9/28/2023). 

## Materials and methods

This study was a biomechanical analysis of four suture permutations using the porcine flexor tendon as a model for the human Achilles tendon.

Specimens

**Figure 1:**
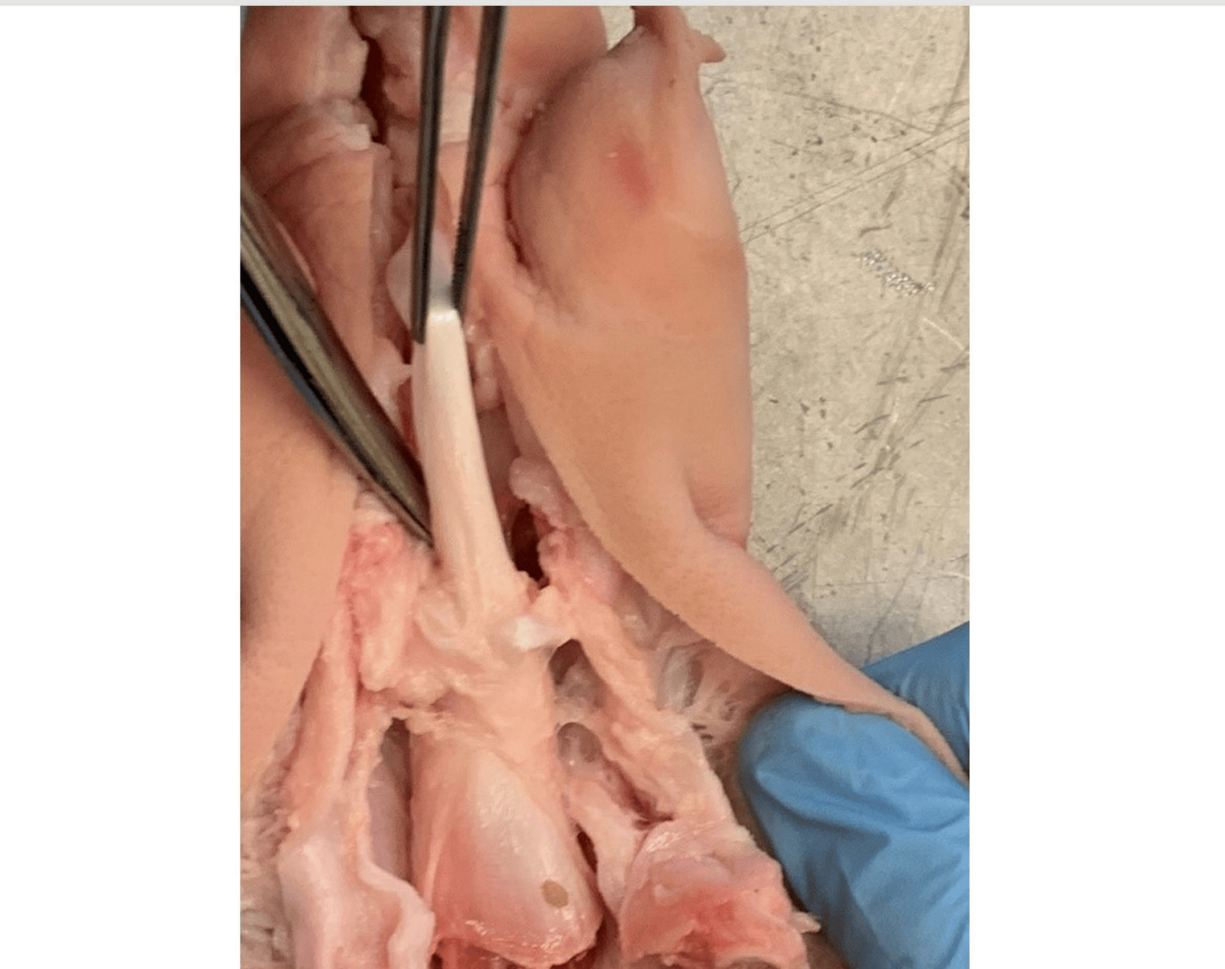
Dissection demonstrating a toe flexor tendon (grasped by forceps) being extracted from a pig trotter.

Porcine toe flexor tendons were used as an analogue for the human Achilles tendon in this study. These have been shown to have similar anatomical and physiological properties to human tendons [7] and have been used in several previous studies that looked at the mechanical properties of Achilles tendon repairs [9-12]. Specimens were excised following the procedure described in Hirpara et al. [9] and underwent visual inspection for any notable problems that may have influenced their behavior (Figure 1). Tendons were wrapped in saline-soaked gauze and stored at -20°C. 

Repair technique

**Figure 2:**
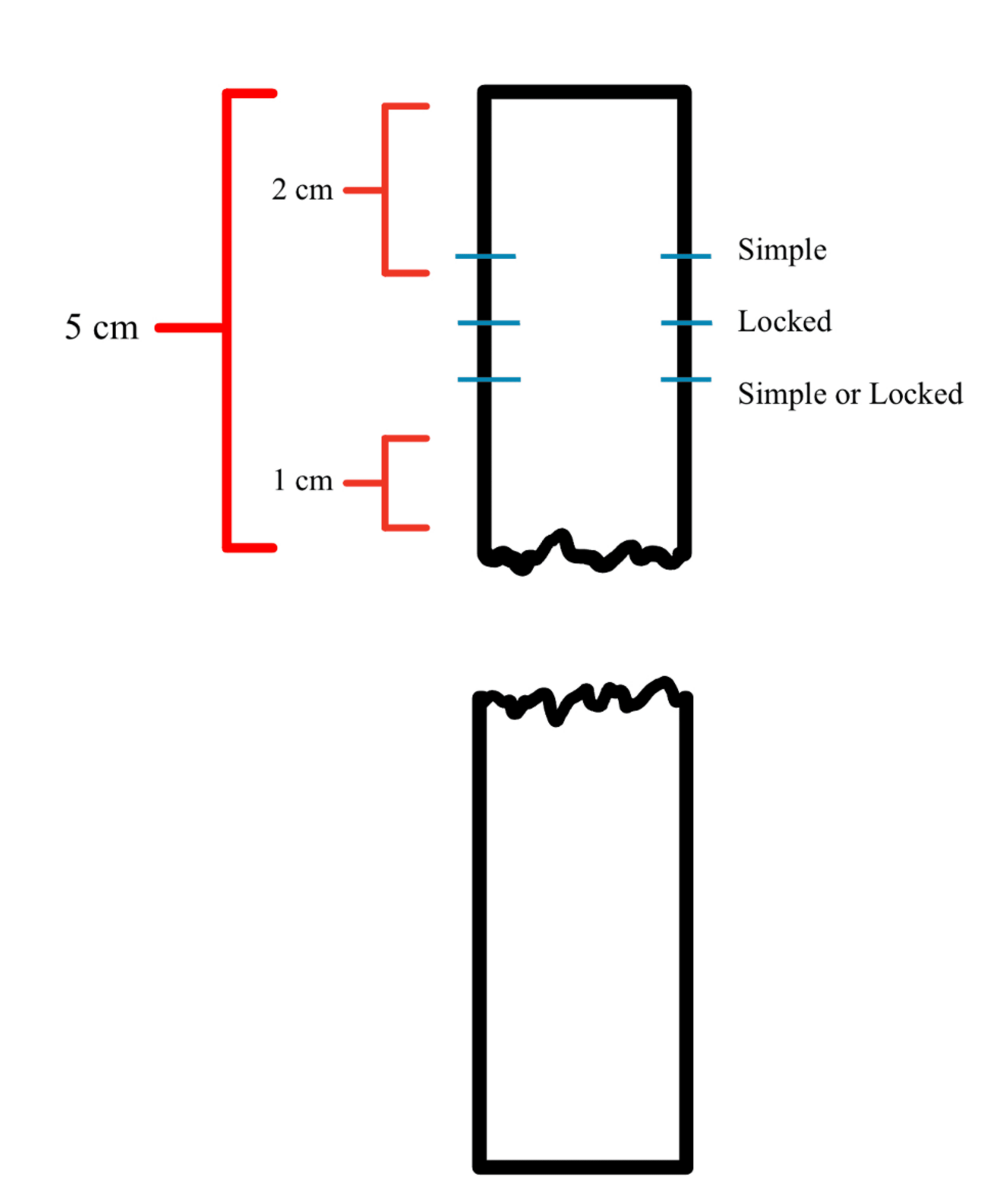
Organization of sutures used in the study This figure has been created by the authors.

**Figure 3:**
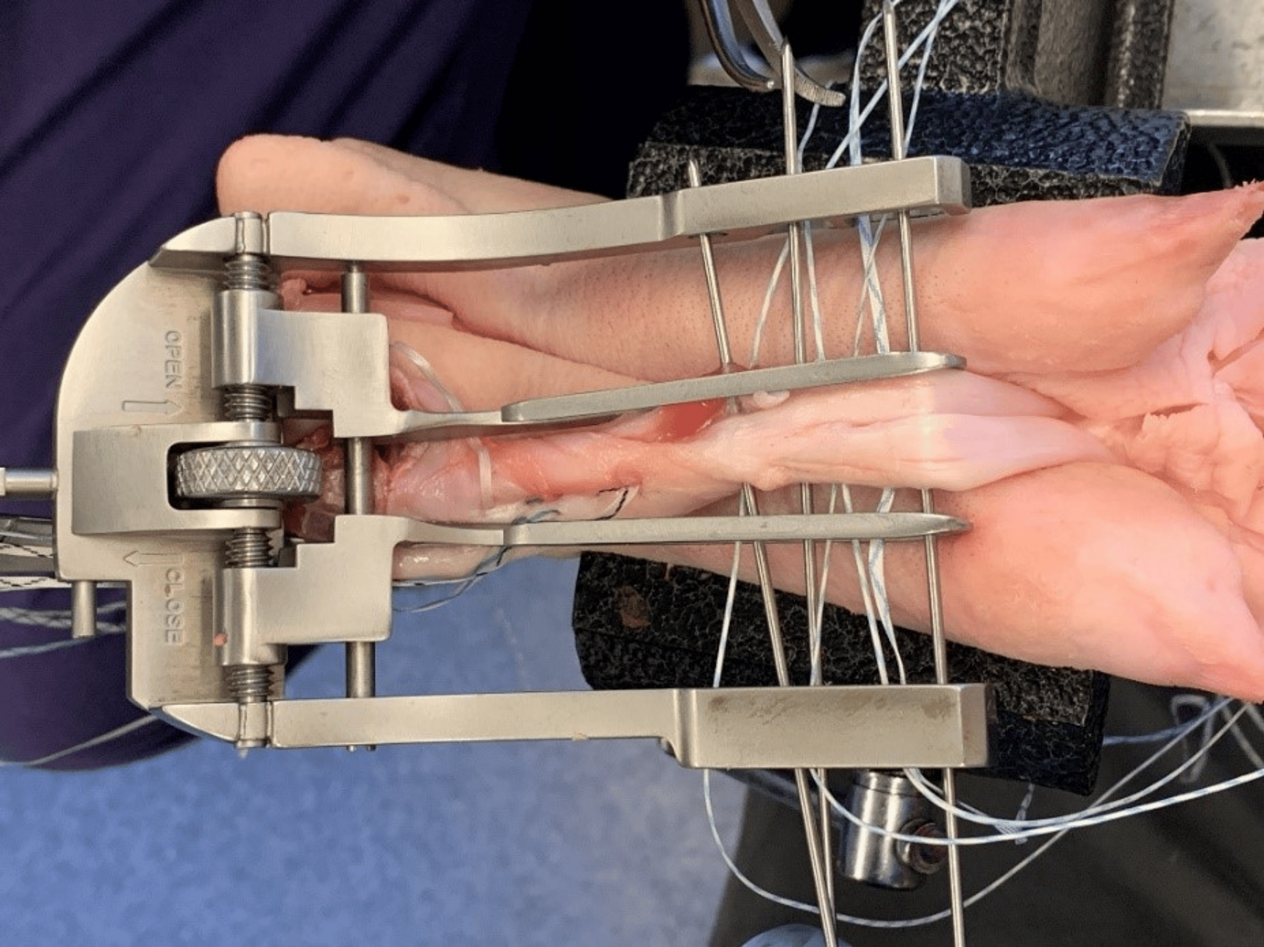
Sutures are passed through a commercially available Arthrex™ jig to simulate passage through the human Achilles tendon.

After extraction, the tendons were sutured according to a pre-determined protocol, as seen in Figure 2. Using a commercially available Arthrex™ (Arthrex®, Inc., Naples, FL) jig, the first suture was positioned to allow 1 cm of space between the cut end of the tendon and the first suture in the configuration (Figure 3). The suture closest to the cut end of the tendon was either a simple (single locked) or a locked suture (double locked). The middle suture was always a locked suture. The suture furthest from the cut end of the tendon was always a simple (unlocked) suture. There was at least 2 cm of tendon between the last suture and the end (non-cut) of the tendon to allow for loading onto the mechanical testing machine. Once sutures were placed in the tendon, it was then cut in half to simulate an Achilles tendon rupture, with each end measuring approximately 5 cm.

The specimens were organized into four groups. They consisted of round suture (#2 Fiberwire™, Arthrex™) with one locked suture (standard), round suture (#2 Fiberwire™, Arthrex™) with two locked sutures, flat (tape) suture (FiberTape™, Arthrex™) with one locked suture, and flat (tape) suture (FiberTape™, Arthrex™) with two locked sutures. A total of 40 tendon repair constructs were randomly assigned to each test group (10 in each). 

Preparation

Prior to testing, tendons were thawed for 30 minutes in a 37°C bath of sodium chloride solution. 

Mechanical testing

**Figure 4:**
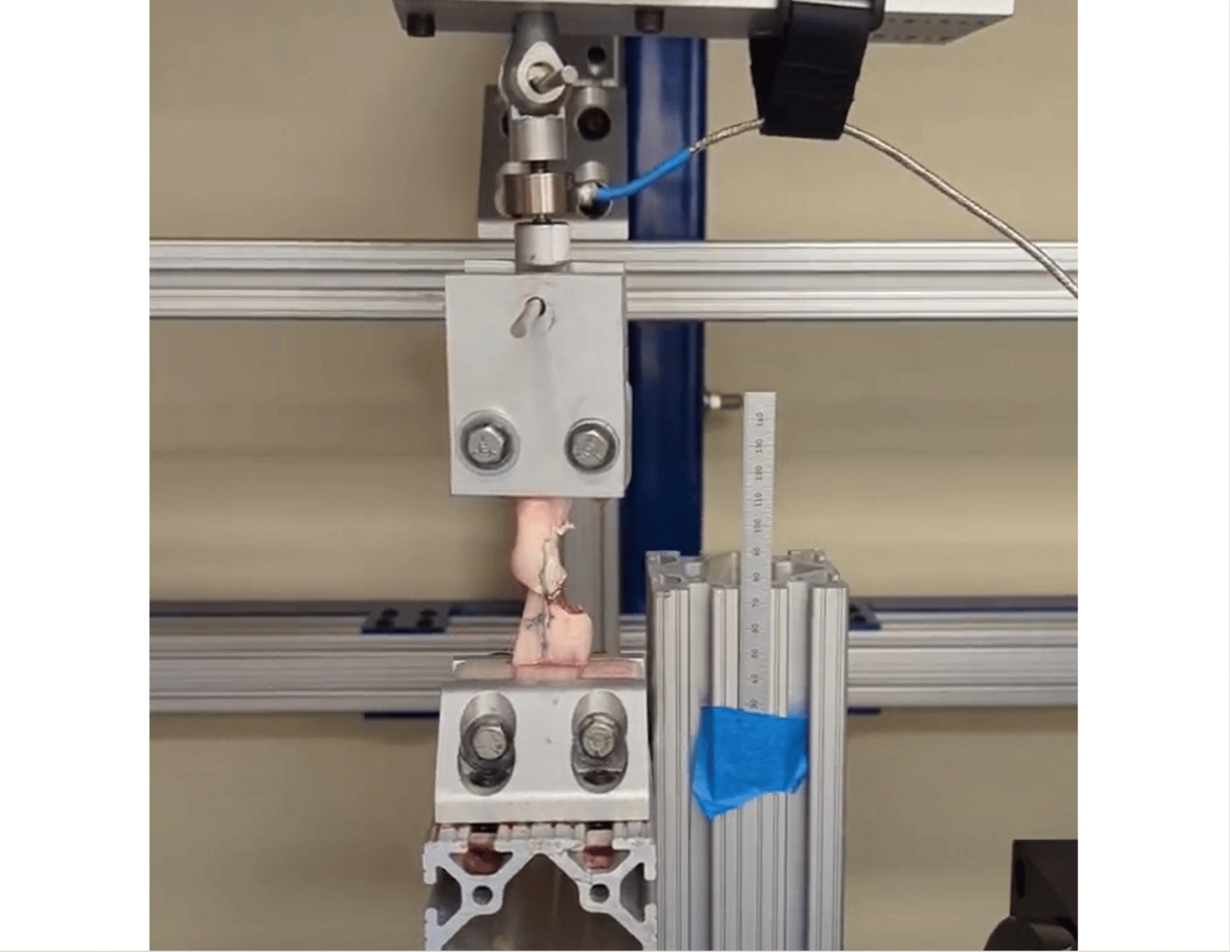
Demonstration of specimen in the testing machine (R2000; Mikrolar Inc.)

Specimen constructs were uniaxially attached to a material testing machine (R2000; Mikrolar, Inc., Hampton, NH) (Figure 4) by mounting the proximal and distal ends in 3D printed clamps, adapted from the design described by Wood et al. [12]. Preliminary testing for this study found these to be reliable and simple to use for the tests performed, and there were no indications of slippage during the failure tests. Clamps were placed 5 mm from the most distal and proximal stitch on the specimen construct. After mounting the specimen, a 1N static load was applied, and the cross-sectional area of the tendon was determined by taking anteroposterior and mediolateral measurements using a micrometer and fitting an elliptical shape defined by these measurements. 

The testing protocol largely followed that described in Jahnke et al. [10] and consisted of a static creep test, a dynamic load creep test, and finally, a load-to-failure test. To determine the static creep behavior of the construct, a load of 30 N was applied for 15 minutes. Cyclic tests were performed at 0.4 Hz for 500 cycles and were defined by a 5 N to 30 N triangular waveform. Visual inspection of the specimen was performed at the end of each of the creep tests to assess if any gaping had occurred. The ultimate failure test was performed at 20 mm/s. There was a 30-minute pause between tests and a 90-second preload of 10N prior to each test. 

Data processing

For the static creep tests, the average strain (%) was determined during the first 10 seconds of the test (after the load had stabilized to within 0.5 N of the target load) and the final 10 seconds of the test, and the difference was calculated. In the case of the dynamic creep test, the maximum strain averaged across the first 10 cycles and final 10 cycles was determined, and the difference was calculated. For the load-to-failure (tear-off) test, the maximum force (N), maximum stress (N/mm^2), and stiffness (N/mm) were determined. 

Statistical analysis

All data analysis was carried out using R (v4.2.2; R Core Team, 2022) [13]. Initially, after confirming the normality of the dataset, an analysis of variance test was performed to assess if the repair type had a significant effect on the mechanical properties of the tendon-repair construct, with the biomechanical variables modeled as the dependent variable and the repair type as the independent variable. This was followed by pairwise comparisons using independent t-tests to assess inter-group differences if significant effects were found, adjusted for multiple comparisons (based on a global significance level of ɑ = 0.05). 

## Results

The results for one specimen (double round group) were removed from the analysis due to failure of the tendon body rather than the stitching construct during the load-to-failure test. 

Static creep test 

There were no significant differences found for strain measurements during the static test (F(3,35) = 0.07, p = 0.95). No gap formation was seen in any of the specimens after this test. 

Cyclic creep test 

There were no significant differences found for strain measurements during the cyclic creep tests (F(3,35) = 0.66, p = 0.58). As with the static test, no gap formation was seen for any of the specimens. 

Load-to-failure test 

**Figure 5:**
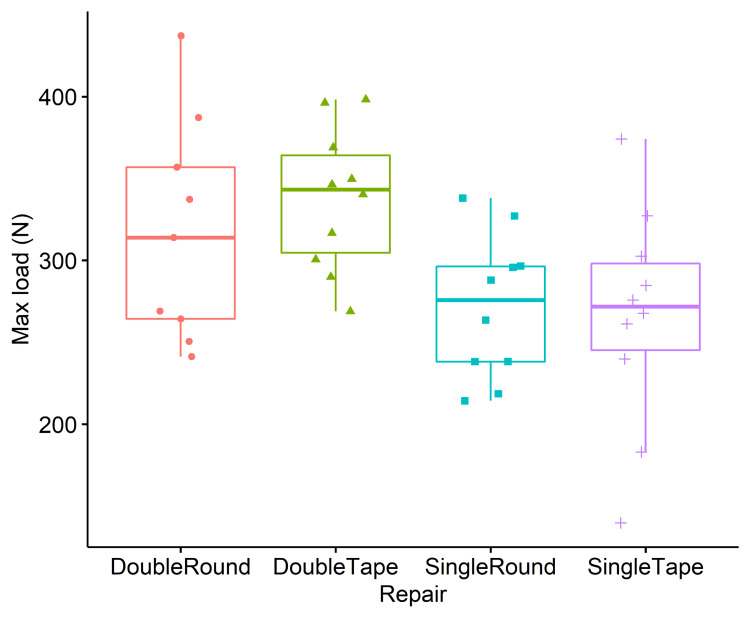
Load-to-failure results Pairwise comparisons demonstrate that double tape had a significantly greater load to failure than single tape and single round conditions. No significant differences were found between the double tape and double round techniques, nor between the double round and either of the single techniques.

The repair used had a significant effect on the maximum load to failure of the constructs (F(3,35) = 3.8, p = 0.018). Pairwise comparisons (Figure 5) showed that double tape had a significantly greater load to failure than single tape and single round conditions (Table 1). No significant differences were found between the double tape and double round techniques, nor between the double round and either of the single techniques. 

**Table 1:** Comparisons for load-to-failure and maximum stress The repair used had an effect on the maximum load to failure of the constructs. Double tape had a significantly greater load to failure than single tape and single round conditions.

Comparison	Difference in maximum load (p, 95% CI)	p Value	95% Confidence interval	Difference in maximum stress (p, 95% CI)	p Value	95% Confidence interval
Double round vs. Double tape	-20 N	0.552	(-77, 37)	-0.03N/mm^2	0.59	(-0.1, 0.05)
Double round vs. Single round	46 N	0.17	(-11, 103)	0.07N/mm^2	0.15	(-0.01, 0.16)
Double round vs. Single tape	52 N	0.17	(-14, 117)	0.07N/mm^2	0.15	(-0.02, 0.16)
Double tape vs. Single round	66 N	0.021	(25, 107)	0.1N/mm^2	0.04	(0.03, 0.17)
Double Tape vs. Single tape	72 N	0.037	(18, 126)	0.1N/mm^2	0.04	(0.02, 0.18)
Single Round vs. Single tape	6 N	0.81	(-48, 60)	0.0N/mm^2	0.995	(-0.08, 0.08)

**Figure 6:**
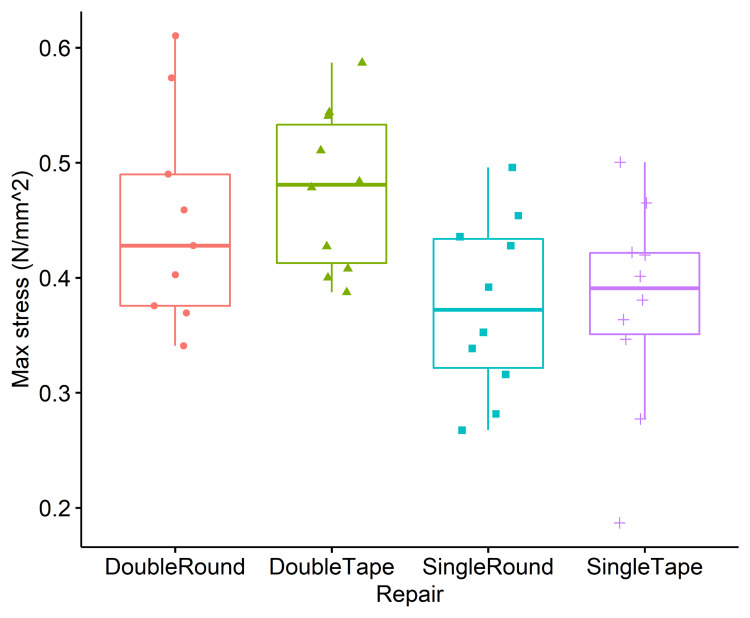
Stress at failure results Pairwise comparisons showed that the double tape repair reached greater stresses before failure compared to the single tape and single round conditions. No significant differences were found between the double tape and double round techniques, nor between the double round and either of the single techniques.

Similarly, the repair had a significant effect on the maximum stress in the construct during the load-to-failure test (F(3,35) = 3.81, p = 0.019). Pairwise comparisons (Figure 6) showed that the double tape repair reached greater stresses before failure compared to the single tape and single round conditions (Table 1). No significant differences were found between the double tape and double round techniques, nor between the double round and either of the single techniques. No significant differences were found for the effect of repair on the overall construct stiffness during the load-to-failure tests. 

## Discussion

Patients who sustain an acute rupture of the Achilles tendon often have symptoms of pain and weakness, and for the athlete, this can result in removal from sport for an average of six months [14]. Currently, there is no compelling data to support surgical over non-surgical management of this injury. The comparison of operative versus nonoperative management for Achilles tendon ruptures is beyond the scope of this study. However, by patient or surgeon preference, many patients undergo operative intervention, and there has been a general trend towards minimally invasive surgery in the United States [15]. Acute re-rupture rates are low [16], but can result in a repeat attempt at repair, causing a delay in recovery, increased cost, and delayed return to sport. This study attempts to understand if suture configuration plays a role in the overall strength of the construct placed at the time of surgery. The results suggest that the use of a double-locked tape suture configuration leads to a stronger overall construct by using a porcine model to simulate a minimally invasive technique for Achilles tendon repairs.

This study has limitations. Although the use of porcine tissue as a surrogate for the human Achilles tendon has been validated in previous biomechanical studies, there may be some limits to the generalizability to in vivo use. The biologic effects, such as healing, human tissue, and suture breakdown during the course of postoperative rehabilitation, cannot be assessed with this method. Additionally, we chose a minimum distance between the cut (simulated ruptured) end of the tendon and the location of our first suture. In a surgical patient, the location of the rupture may dictate the amount of tendon available to suture.

In our experience, the use of a double-locking suture construct (compared to a single one) adds little time to the surgical treatment of Achilles tendon ruptures and can add significant strength to the surgical repair at time 0. Theoretical disadvantages include an increased number of percutaneous punctures into the skin and tendon itself and a possible increased risk of sural nerve injury, although this has not been studied. Further clinical studies are needed, but a stronger repair construct may lead to a decreased re-rupture rate or decreased amount of tendon elongation. We suggest considering using this construct in patients who may be at higher risk for re-rupture, such as the competitive athlete or patients who are expected to put increased stress on the repair early in their recovery. 

## Conclusions

Although surgical management of the Achilles tendon rupture is often performed, little is known about the biomechanics of the sutures used or the difference between different suture configurations. Many commercially available jigs for this injury, including the one used in this study, have built-in extra guide holes for additional suture. To our knowledge, no study has been published about the biomechanics of the use of additional suture, additional locked suture, or the use of flat versus round suture.

## References

[1] Doral MN, Alam M, Bozkurt M, Turhan E, Atay OA, Dönmez G, Maffulli N (2010). Functional anatomy of the Achilles tendon. Knee Surg Sports Traumatol Arthrosc.

[2] Demetracopoulos CA, Gilbert SL, Young E, Baxter JR, Deland JT (2014). Limited-open Achilles tendon repair using locking sutures versus nonlocking sutures: an in vitro model. Foot Ankle Int.

[3] Ganestam A, Kallemose T, Troelsen A, Barfod KW (2016). Increasing incidence of acute Achilles tendon rupture and a noticeable decline in surgical treatment from 1994 to 2013. A nationwide registry study of 33,160 patients. Knee Surg Sports Traumatol Arthrosc.

[4] Zellers JA, Carmont MR, Grävare Silbernagel K (2016). Return to play post-Achilles tendon rupture: a systematic review and meta-analysis of rate and measures of return to play. Br J Sports Med.

[5] Sheth U, Wasserstein D, Jenkinson R, Moineddin R, Kreder H, Jaglal SB (2017). The epidemiology and trends in management of acute Achilles tendon ruptures in Ontario, Canada: a population-based study of 27 607 patients. Bone Joint J.

[6] Willits K, Amendola A, Bryant D (2010). Operative versus nonoperative treatment of acute Achilles tendon ruptures: a multicenter randomized trial using accelerated functional rehabilitation. J Bone Joint Surg Am.

[7] Reito A, Mattila V, Karjalainen T (2022). Operative vs. nonoperative treatment of Achilles tendon ruptures using early functional rehabilitation: critical analysis of evidence. Foot Ankle Int.

[8] Myhrvold SB, Brouwer EF, Andresen TK (2022). Nonoperative or surgical treatment of acute Achilles’ tendon rupture. N Engl J Med.

[9] Hirpara K AO, O’Neill B, O’Sullivan M (2006). A technique for porcine flexor tendon harvest. J Musculoskelet Res.

[10] Jahnke A, Gernandt M, Hudel H, Ahmed GA, Rickert M, Fonseca Ulloa CA, Stolz D (2019). Biomechanical testing of various suture techniques for Achilles tendon repair with and without augmentation by using synthetic polyester grafts. J Biomech.

[11] Mao WF, Wu YF, Zhou YL, Tang JB (2011). A study of the anatomy and repair strengths of porcine flexor and extensor tendons: are they appropriate experimental models?. J Hand Surg Eur Vol.

[12] Vella Wood M, Casha A, Gatt A (2019). 3D printed clamps to study the mechanical properties of tendons at low strains. Phys Status Solidi B.

[13] Team RC (2019). The R Project for Statistical Computing. R: A language and environment for statistical computing.

[14] Ochen Y, Beks RB, van Heijl M (2019). Operative treatment versus nonoperative treatment of Achilles tendon ruptures: systematic review and meta-analysis. BMJ.

[15] Ko PY, Hsu CH, Hong CK (2021). Jigless knotless internal brace versus other open Achilles tendon repairs using a progressive rehabilitation protocol: a biomechanical study. BMC Musculoskelet Disord.

[16] Mattingly AS, Chen MM, Divi V, Holsinger FC, Saraswathula A (2023). Minimally invasive surgery in the United States, 2022: understanding its value using new datasets. J Surg Res.

